# How to maintain underground social relationships? Chemosensory sex, partner and self recognition in a fossorial amphisbaenian

**DOI:** 10.1371/journal.pone.0237188

**Published:** 2020-08-19

**Authors:** José Martín, Ernesto Raya García, Jesús Ortega, Pilar López

**Affiliations:** 1 Departamento de Ecología Evolutiva, Museo Nacional de Ciencias Naturales, CSIC, Madrid, Spain; 2 Laboratorio de Herpetología, Instituto de Investigaciones sobre los Recursos Naturales, Universidad Michoacana de San Nicolás de Hidalgo, Morelia Michoacán, México; 3 Department of Biology, Lund University, Lund, Sweden; Institute of Animal Science, CZECH REPUBLIC

## Abstract

Maintaining social relationships depends on the ability to recognize partners or group members against other individuals. This is especially important in animals with relatively stable social groups. The amphisbaenian *Trogonophis wiegmanni* is a semi blind fossorial reptile that spends its entire life underground where it interacts with mates and social partners. In this environment, visual cues are limited. Chemosensory cues may rather allow conspecific social and partner recognition. We recorded the number of tongue-flick (TF) rates of *T*. *wiegmanni* amphisbaenians to scents of both sexes with different pairing social bonds (familiar vs. unfamiliar) presented on cotton swabs to test discrimination of social groups. As seen from a rise in the number of TFs, males discriminated unfamiliar females from unfamiliar males. This suggests that chemical cues may be used by males to locate new mates. In contrast, females detected scent of unfamiliar conspecifics, but did not show sex discrimination. Both males and females discriminated the scent of an individual with which they had formed a pair bond from an unfamiliar individual of the same sex as the partner. Also, males, but not females, were capable of self-recognition, suggesting that scent marks of males in home ranges may provide individual information in intrasexual relationships. We conclude that conspecific discrimination based on chemical cues may allow the maintenance of social relationships and relatively stable pairs in fossorial reptiles inhabiting visually restricted environments.

## Introduction

Whether animals can maintain cohesive and long-term relationships depends on their ability to distinguish between partners, familiar or related individuals, or group members from other unfamiliar individuals [e.g. 1–5]. Reptiles rarely show stable pair and social aggregations [but see 6–9]. Viviparity seems to be a relevant factor in the evolution of sociality in reptiles [10]. Nevertheless, in at least some viviparous reptile species we see the occurrence of social cohesive groups (e.g. Australian skinks of the genera *Tiliqua* and *Egernia*, [8, 9, 11, 12]). In these skink species, pairs and family groups typically remain stable for several seasons and adults jointly/cooperatively defend their home range against other conspecifics [13, 14]. Even in lizard species that are not viviparous and do not form long term groups, recognition of the partner against other individuals of the same sex may allow prolonged mate guarding and maintenance of stable pair relationships with a given partner at least during one mating season [15–19].

In lizards, conspecific recognition abilities needed to maintain social links are often based on chemical cues [e.g. 20–22; reviewed in 23, 24] (although the use of additional cues has not been discounted [25]). Chemosensory recognition by males of the scent of different individual females may facilitate both mate guarding of the female partner and the locating new female mates (as it occurs in the snow skink, *Niveoscincus microlepidus* [17] or the broad headed skink, *Eumeces laticeps* [15, 16]). Similarly, familiarity with the male partner affects to the trend of a female sleepy lizard (*T*. *rugosa*) to follow his scent-marked path [18].

Amphisbaenians are a major distinctive group of fossorial reptiles. Little is known about their ecology and social behavior because studying them is difficult given their secretive habits [26, 27]. Amphisbaenians have evolved morphological traits for living underground, such as reduced vision and the loss of limbs [26–29]. These adaptations can affect the behavioral responses of fossorial amphisbaenians, which may greatly differ from those of epigeal reptiles [e.g. 30–35]. One problem that fossorial animals face is how to identify and interact with conspecifics. The use of visual cues is clearly limited by the fossorial environment and by the rudimentary vision [26]. Therefore, chemoreception may be the prominent sensory modality for conspecific communication. Previous studies have shown that at least one species of amphisbaenian, *Blanus cinereus*, uses chemical cues in sex discrimination and self-recognition [36–38], and for scent-marking their home ranges [39].

The amphisbaenian *Trogonophis wiegmanni* spent its entire life buried in sandy soils and under rocks [40, 41]. This amphisbaenian is capable of using chemical cues to identify their prey types [42]. It is a viviparous species [43] that, interestingly, is often observed in pairs or seemingly stable “family groups” under the same rock [44], suggesting that at least some relatively long-term pair bonding exists in this species. However, the importance of these social pair aggregations, and whether and how partner recognition occurs are unknown. We hypothesized that detailed chemosensory discrimination of conspecifics should be found in fossorial reptile species, such as *T*. *wiegmanni*, because of its importance in the maintenance of stable pairs or social relationships in these animals.

In this paper, we tested whether *T*. *wiegmanni* amphisbaenians are able to detect and discriminate between different classes of conspecifics using information from chemical cues. We specifically examined whether adult amphisbaenians can: a) detect chemical cues from conspecifics, b) determine signaler sex, c) discriminate between a familiar partner and an unfamiliar individual of the same sex, and d) discriminate between self and conspecific odors. We discuss how chemosensory discrimination between conspecifics may contribute to maintaining social relationships in fossorial animals.

## Material and methods

### Study animals

The amphisbaenian *T*. *wiegmanni* is a northwestern African Mediterranean species found from Morocco to northeast Tunisia [45]. Similarly to other amphisbaenians, the knowledge of its ecology and behavior is limited, but increasingly more studies are providing information on its habitat use [40, 41], diet and prey detection [42, 43, 46], thermal biology [47, 48], reproductive cycle [42], and population structure [44, 49, 50].

We captured live amphisbaenians in April 2012 at the Chafarinas Islands (Spain), a small volcanic archipelago located in the southwestern area of the Mediterranean Sea (35°11’N, 2°25’W). On these islands, *T*. *wiegmanni* is very abundant [51]. We walked around the islands between 07:00 and 18:00 (GMT) lifting stones under which active amphisbaenians were typically found [48]. When we found a pair of amphisbaenians close together under the same rock (*N* = 15 pairs; snout-to-vent length, SVL, mean ± SE = 148 ± 4 mm), we captured both individuals by hand. Sex was determined by carefully trying to evert the hemipenes from the cloacas of males [44, 49, 50]. In cases where two adults were found together (often in close body contact) one was a male and one was a female. Individuals found alone were not captured.

Pairs of amphisbaenians were carefully transported to the laboratory in separate plastic boxes (one for each pair) with sand from their respective capture sites following recommended procedures [52]. Amphisbaenians were housed at “El Ventorrillo” MNCN-CSIC Field Station (Navacerrada, central Spain). The field pair relationships were maintained in captivity throughout the whole experiment by keeping together each pair of amphisbaenians in an indoor plastic terrarium (40 x 30 x 30 cm) with a loose 5 cm depth coconut fiber substrate. Sunlight with a natural photoperiod entered into the room through two large windows, although amphisbaenians were never observed exposed above the substrate surface. We placed below the terraria a heating cable, connected to a thermostat, which allowed amphisbaenians to achieve an optimal temperature by thigmothermy with the warm substrate (around 24–25 °C) [47, 48]. Each terrarium had a flat tile (20 x 20 cm) placed above the substrate as a shelter under which amphisbaenians could thermoregulate and forage [53, 54]. To feed amphisbaenians, we placed three times per week mealworm larvae and pupae, as well as freshly killed crickets, dusted with a multivitamin powder, underneath each tile [54]. Food was readily eaten by amphisbaenians within a few hours. The substrate was moistened every day with a water spray to avoid desiccation and to provide drinking water. All amphisbaenians were healthy during this study and maintained an optimal body condition. All work detailed here was approved by the Spanish National Parks Authority and the Ethical Committee of the Museo Nacional de Ciencias Naturales, CSIC.

### Chemosensory tests

To assess detection and discrimination of chemical cues of conspecifics, we quantified the tongue-flick (TF) behavior of amphisbaenians as an indirect measure of the chemosensory response to different chemical stimuli presented on cotton swabs. This method is based on the assumption that discrimination of the different chemical stimuli is indicated by differences in TF responses [36, 55–57]. We compared the TF rates of amphisbaenians to scents of different categories of conspecifics in different experiments. We used deionized water as a control to assess the baseline TF rates in the experimental setup [55].

The pairs of amphisbaenians used in this study were found at well-separated field sites (more than 50 m between capture sites) and the underground dispersal ability of amphisbaenians is very low (changes in location between successive recaptures in different years and seasons are on average of about 4 m inside home ranges of about 6 m^2^; unpubl. data). Thus, we assumed that individuals of each pair had not had contact with individuals from the other pairs tested here and were considered as unfamiliar individuals, whereas individuals within each pair were considered as familiar individuals.

We conducted trials during June, when the amphisbaenians were fully active (between 1100 h and 1500 h GMT). To prepare the scent stimuli, just prior each tests, we first dipped the cotton tip (1 cm) of a wooden applicator (10 cm) in deionized water and then we rolled the moistened cotton over the cloaca of the donor amphisbaenian to add its scent stimuli. We used a new swab with a new stimulus in each trial.

Before the experiments, we gently transferred the responding animal from its home terrarium to a small clean cage (20 x 15 cm) that contained a 0.5 cm depth clean coconut substrate, and left them for 15 min for acclimation to the new cage before tests. Amphisbaenians remained semi-buried in the substrate, which allowed to observe their responses while they behave normally (i.e. amphisbaenians did not show signs of stress such as quick escape locomotion or freeze defensive behavior). Trials were conducted in partial darkness and using a red light to avoid disturbing amphisbaenians. Because tongue flick rates depends on body temperature in lizards [58], we maintained room temperature at 24 °C, to allow amphisbaenians to attain their preferred body temperature [47, 48].

All trials were conducted by the same experimenter, (PL) who was blind to the treatments. In each trial, we slowly positioned the cotton swab with the stimulus 1 cm in front of the head of the amphisbaenian, which, in all tests, responded to the scent stimuli by tongue-flicking the swab. We tested each amphisbaenian once per day with one stimulus and tested the rest of the scent stimuli in subsequent days in a counterbalanced order. After each test, we immediately returned each amphisbaenian to its home terrarium and cleaned the experimental cage with water and soap to remove any amphisbaenian chemicals. Several identical experimental cages with new coconut substrates were used for different tests. We let a week between experiments to allow amphisbaenians to rest.

In a first experiment, we used a discrimination test to examine the existence of chemosensory conspecific detection and sex discrimination by *T*. *wiegmanni* amphisbaenians. We compared the chemosensory responses of adult amphisbaenians to scents of a) water as a clean control, b) an unfamiliar male and c) an unfamiliar female, neither of which was the partner of the responding individual. We considered that higher TF rates to conspecific scent than to water would indicate chemosensory conspecific detection and that differential TF rates to male and female stimuli would indicate sex discrimination.

A second experiment was designed to test for familiar scent recognition using a classical habituation-dishabituation procedure [59–62]. In this technique, an individual is first habituated to an odor presented in repeated occasions, and subsequently a new odor is introduced in the dishabituation phase. If this new odor was discriminated from the previous one, we should observe a change in the responses. We tested the chemosensory responses of amphisbaenians in two treatments: a) water (as a clean control of the experimental setup), in both the habituation and dishabituation trials, and b) scent of the familiar partner paired with the responding amphisbaenian in the habituation trials, followed in the dishabituation trial by scent of an unfamiliar individual of the same sex than the partner and of similar body size/age that had never been in contact with the responding amphisbaenian. Responding individuals were tested in the two treatments in different days in a counterbalanced order.

In the three habituation trials, we presented each amphisbaenian with a cotton swab with either water (control) or scent of its partner, repeating this process three times. In each trial, we presented the same swab and recorded the numbers of TF directed toward the swab for a duration of 60 s, following the initial TF. Then, we removed the swab, waited for 1 min and presented the same swab again to record the second TF rates, and did the same for the third TF rates. Immediately following these three habituation trials, we waited for 1 min and conducted one dishabituation trial, presenting a new cotton swab with either water (control) or scent of an unfamiliar male or female (for responding females and males respectively). We predicted that, if amphisbaenians were able of familiar or partner chemosensory discrimination, the TF rates in the last habituation trial and the dishabituation trial should be different, but TF rates should not differ in the water (control) treatment.

In a third experiment, to test for self recognition in adult male and female amphisbaenians, we used a similar habituation–dishabituation procedure as above. Here each male or female amphisbaenian participated in a counterbalanced order in two treatments. Each individual was first tested repeatedly in three habituation trials with the same cotton swab in two treatments with either water (control) or its own scent. Then, we tested each amphisbaenian in one dishabituation trial with a new cotton swab with either water (for the control treatment) or scent of an unfamiliar male or female (for responding males and females respectively) that had never been in contact with the responding individual. We hypothesized that if chemosensory exploration TF rates increased during the dishabituation trials with scent of an unfamiliar individual, this would indicate self-recognition.

### Data analyses

In the first experiment, to test for differences in TF rates of amphisbaenians among chemical stimuli, we used a repeated measures General Linear Model (GLM) with treatment' as a within factor (three levels: water, scent of an unfamiliar male, and scent of an unfamiliar female), and 'sex' of the responding amphisbaenian as a fixed factor, and included the interaction in the model. We log transformed data to ensure normality and homogeneity of variances (checked with Hartley’s Fmax tests). Post-hoc pairwise Tukey’s tests were used to compare TF rates testing for (1) differences among treatments within each sex, and (2) differences between sexes in the responses to the same treatment.

In the second and third experiments, we used repeated measures GLMs with ‘trial’ (four levels: the three habituation trials and the dishabituation trial) and ‘treatment’ (two levels: water and conspecific scent) as within factors, and included the interaction in the models. We analyzed separately the responses of males and females because they responded to different treatments (e.g. males responded only to familiar and unfamiliar females, but not to males, and the converse occurred for responding females). Post-hoc pairwise Tukey’s tests were used to compare TF rates testing for (1) habituation to repeated samples of the same chemical stimuli (comparing responses in the first vs. third habituation trials), and (2) discrimination of the new chemical stimuli (comparing the third habituation trial vs. the dishabituation trial). All analyses were made using Statistica 7.0 software (StatSoft Inc, Tulsa, OK. USA).

## Results

### Conspecific and sex discrimination by male and female amphisbaenians

**Fig 1:**
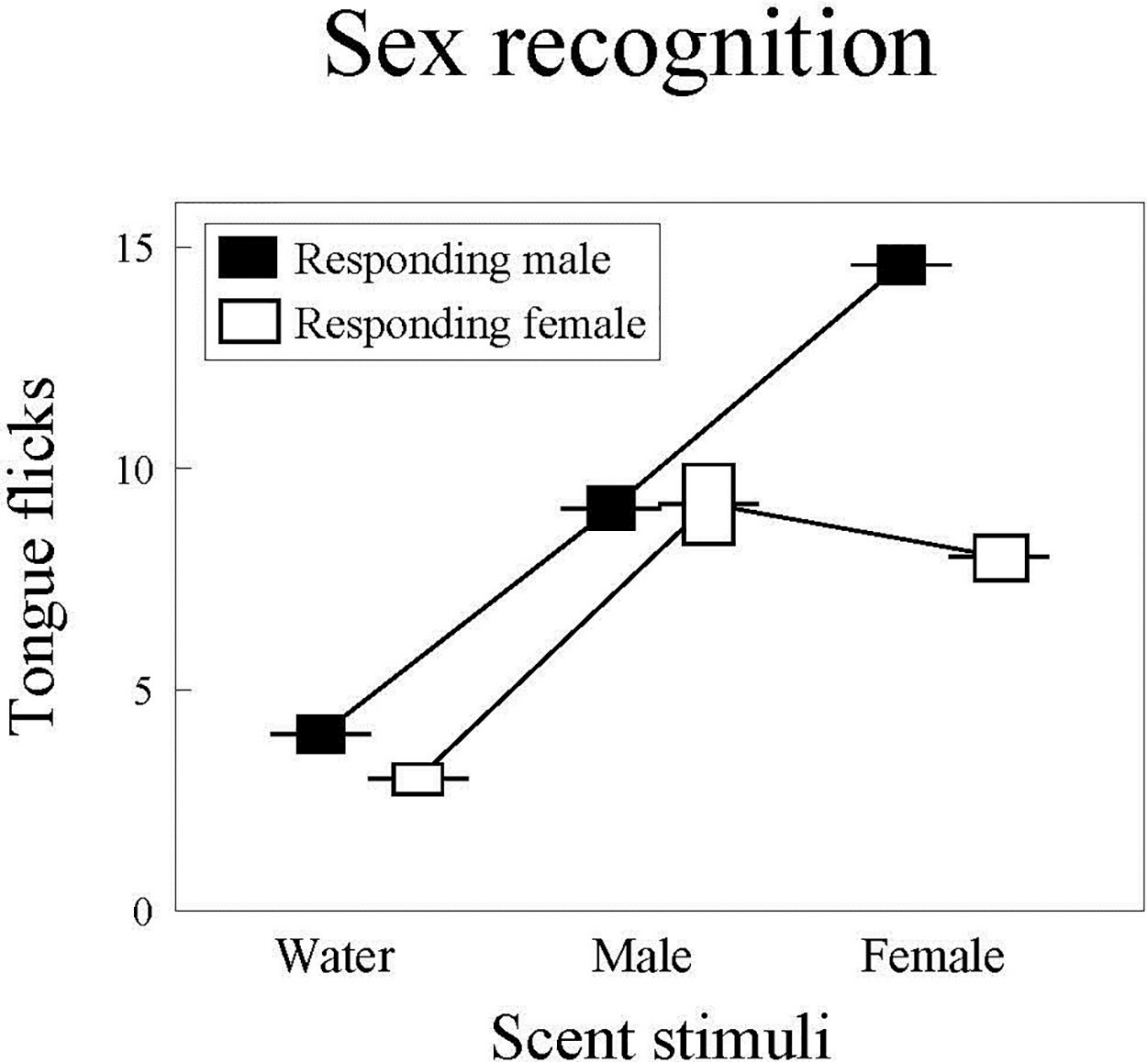
Sex recognition in *T*. *wiegmanni* amphisbaenians. Number (mean ± SE) of directed tongue-flicks emitted by male (open boxes) and female (black boxes) amphisbaenians in 60 sec in response to scent stimuli (water or scent of unfamiliar male or female conspecifics) presented on cotton swabs.

There were significant differences in TF rates of amphisbaenians among treatments (repeated measures GLM, *F*_2,56_ = 162.23, *P* < 0.0001) and between sexes (*F*_1,28_ = 13.50, *P* = 0.001) but the interaction between treatment and sex of the responding amphisbaenian was significant (*F*_2,56_ = 8.51, *P* < 0.0006) (Fig 1).

The post-hoc comparisons among treatments showed that TF rates of males to any conspecific scent were significantly higher than to water (Tukey’s tests, *P* < 0.0002 in both cases), and that TF rates to unfamiliar male scent were significantly lower than to unfamiliar female scent (*P* < 0.0004) (Fig 1). In females, TF rates to any conspecific scent were also significantly higher than to water (*P* < 0.0002 in both cases). TF rates to unfamiliar male and female scents were not significantly different (*P* = 0.87) (Fig 1). Males and females did not significantly differ in their TF rates to water (*P* = 0.32) or scent of unfamiliar males (*P* = 0.99), but responses to scent of unfamiliar females were significantly higher in males than in females (*P* < 0.004).

### Familiar partner recognition by males

**Fig 2:**
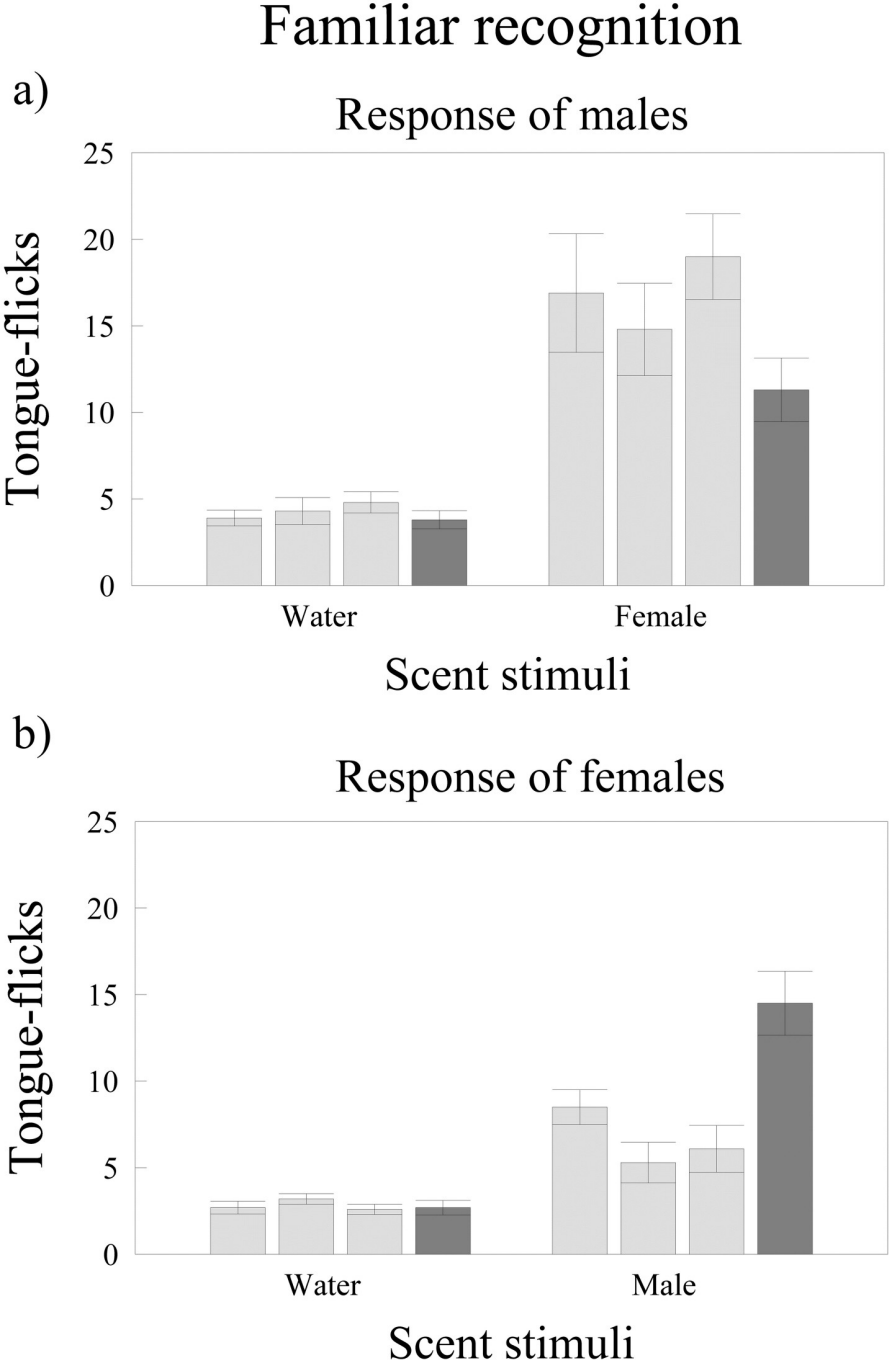
Familiar recognition in *T*. *wiegmanni* amphisbaenians. Number (mean ± SE) of directed tongue-flicks emitted by a) male and b) female amphisbaenians in 60 sec in response to odor stimuli presented on cotton swabs. For each treatment, we made three habituation trials (light grey) with swabs bearing water or scent from the familiar partner (the female or the male of each pair for the responding male or female of the same pair respectively) followed by a dishabituation trial (dark grey) with swabs bearing water or scent of an unfamiliar female or male.

There were significant differences in TF rates of males among trials (repeated measures GLM, *F*_3,27_ = 3.95, *P* = 0.018) and between treatments (*F*_1,9_ = 28.69, *P* < 0.0005) but the interaction between trial and treatment was only marginally significant (*F*_3,27_ = 2.89, *P* = 0.054). Post-hoc tests showed that males had similarly low TF rates in the first vs. the third habituation trials in both the water (Tukey’s tests, *P* > 0.99) and the female treatments (*P* > 0.27) (Fig 2A). However, while in the water treatment there were no significant differences in TF rates between the dishabituation trial and the previous third habituation trial (*P* = 0.99), responses of males to scent of a new individual female in the dishabituation trial were significantly lower than to the scent of his familiar female partner in the previous third habituation trial (*P* = 0.0033) (Fig 2A).

### Familiar partner recognition by females

There were significant differences in TF rates of females among trials (repeated measures GLM, *F*_3,27_ = 11.73, *P* < 0.0001) and between treatments (*F*_1,9_ = 36.16, *P* = 0.0002) but the interaction between trial and treatment was significant (*F*_3,27_ = 11.96, *P* < 0.0001). Females had similarly low TF rates in the first vs. the third habituation trials in both treatments (Tukey’s tests, water: *P* > 0.99; male: *P* > 0.53) (Fig 2B). In the water treatment there were no significant differences in TF rates between the dishabituation trial and the previous third habituation trial (*P* = 0.99). However, responses of females to scent of a new unfamiliar individual male in the dishabituation trial were significantly higher than to the scent of its familiar male partner in the previous third habituation trial (*P* = 0.0012) (Fig 2B).

### Self recognition by males

**Fig 3:**
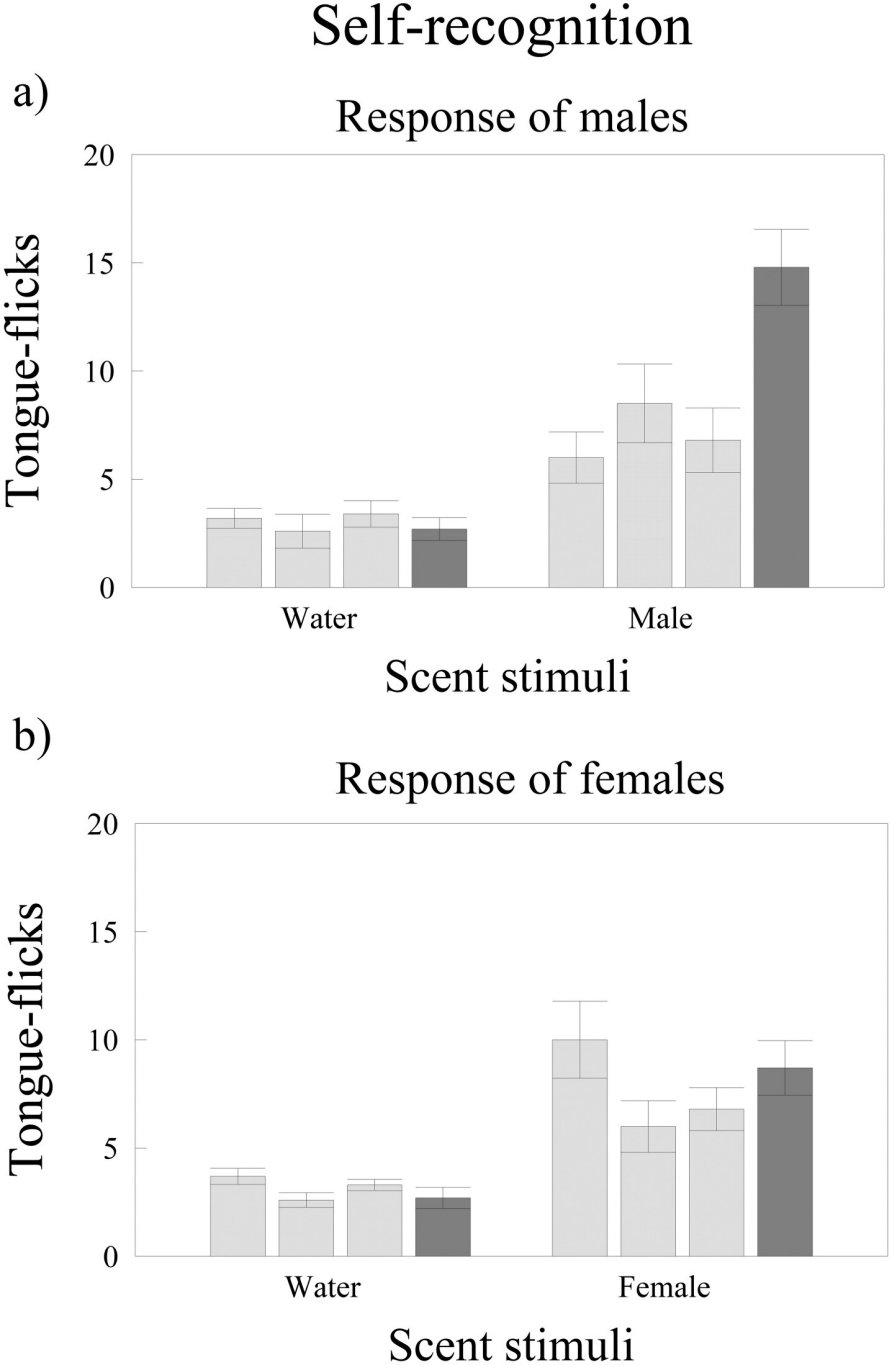
Self recognition in *T*. *wiegmanni* amphisbaenians. Number (mean ± SE) of directed tongue-flicks emitted by a) male and b) female amphisbaenians in 60 sec in response to odor stimuli presented on cotton swabs. For each treatment, we made three habituation trials (light grey) with swabs bearing water or the own scent of the responding amphisbaenian followed by a dishabituation trial (dark grey) with swabs bearing water or scent of an unfamiliar amphisbaenian of the same sex.

The TF rates of males differed significantly among trials (repeated measures GLM, *F*_3,27_ = 6.83, *P* = 0.0014) and between treatments (*F*_1,9_ = 28.90, *P* < 0.0005) but the interaction between trial and treatment was significant (*F*_3,27_ = 9.21, *P* = 0.0002). Post-hoc tests showed that males had no significant differences in TF rates comparing the first vs. the third habituation trials in both the water and their own scent treatments (Tukey’s tests, *P* = 0.99 in both cases) (Fig 3A). However, while in the water treatment there were no significant differences in TF rates between the dishabituation trial and the previous third habituation trial (*P* = 0.99), responses of males to scent of other individual male in the dishabituation trial were significantly higher than to their own scent in the previous third habituation trial (*P* = 0.00024) (Fig 3A).

### Self recognition by females

There were significant differences in TF rates of females among trials (repeated measures GLM, *F*_3,27_ = 3.96, *P* = 0.018), females showed lower overall TF rates in the water treatment (*F*_1,9_ = 22.18, *P* = 0.0011) and the interaction between trial and treatment was not significant (*F*_3,27_ = 2.04, *P* = 0.13) (Fig 3B), Post-hoc tests for the trials main effect showed that females had similar TF rates in the first vs. the third habituation trials (Tukey’s tests, *P* = 0.11) and that there were no significant differences between the dishabituation trials and the previous third habituation trials (*P* = 0.83) (Fig 3B).

## Discussion

Our results are consistent with the hypothesis that the amphisbaenian *T*. *wiegmanni* is able to discriminate scent of several categories of conspecifics using chemical cues. At least male amphisbaenians are able to recognize sex of unfamiliar conspecifics based on scent alone. Moreover, both males and females can discriminate between a familiar partner and an unfamiliar individual of the same sex as the partner. Finally, males, but not females, are also capable of self-recognition.

With respect to sex and familiar recognition, adult males discriminated between scent of unfamiliar males and females. For males that found a conspecific or its scent marks, it should be important to discriminate quickly potential male rivals, which might show aggressive responses, from potential mates (i.e. females). This discrimination might be possible if chemical cues found in the cloacal secretions of males and females were different (something that remains to be tested with further chemical analyses). Similarly, another amphisbaenian species, *Blanus cinereus*, is able of chemosensory sex discrimination [36], which is based on differences in compounds (particularly differences in the concentration of squalene) secreted by the precloacal pores [38].

Also, males discriminated and showed higher chemosensory responses toward their female partners than to unfamiliar females. It may be important for males to discriminate between different individual females if males can copulate with different individual females. Thus, finding an unfamiliar female may provide the opportunity to obtain additional matings to those that the male could presumably had already obtained with the familiar female that shared his home range (i.e. the terrarium in the experimental situation). This would explain the differential chemosensory rates to familiar and unfamiliar females. Chemosensory recognition by males of scent marks of different individual females may facilitate both maintaining pair bonding with a particular female and the location of new females. Thus, given that this amphisbaenian seems to form stable pairs of long-term duration [44], familiar chemosensory recognition may allow mate guarding of a specific female partner, as it occurs in the snow skink, *Niveoscincus microlepidus* [17], or in the broad headed skink, *Eumeces laticeps* [15, 16]. Similarly, male leopard geckos (*Eublepharis macularius*) are able to become familiar with two different females, and discriminate between them [62].

In contrast, although females clearly detected conspecific scent and discriminated it from a blank control, females did not show differential responses to scent of unfamiliar males and females. This was an unexpected result as females of the amphisbaenian *B*. *cinereus* also discriminate between sexes [38]. It is possible that females do not need to discriminate between sexes of unfamiliar individuals, or do not show different responses, if females just need to detect that a conspecific is nearby. Sex discrimination might not be important if females were not aggressive with other females and males were neither a threat for a female. However, females clearly discriminated between their familiar male partner and an unfamiliar male. Therefore, discrimination of the male partner may be important to maintain the pair cohesion and to avoid being harassed by other unfamiliar males. Females might also be interested in detecting unfamiliar males as a potential source of extrapair matings. Similarly, in social lizards, females seem to recognize the scent marks of their male partners and use this information to maintain pair bondings [18].

With respect to self-recognition, males, but not females, seem capable of discriminating between their own odor and the odor of an unfamiliar individual of the same sex. Similar behaviors have been reported in males of the amphisbaenian *Blanus cinereus* [37] and in several lizard species [63–68]. This ability of discrimination would be especially important for males to rapidly detect and recognize potential competitors (or their scent marks), with which costly agonistic encounters could occur [20, 69]. In contrast, for females, similar to many animals, the encounter between two individual females would not usually result in any aggressive interaction [23; but see 70]. In contrast, self-discrimination in females occurs in other lizard species, as in *Liolaemus tenuis*, because it might be important for establishing of female hierarchies inside a male territory [67, 71]. Our results may suggest that the ability to discriminate between self-scent marks and those of other individuals of the same sex could have evolved only in male *T*. *wiegmanni*, while self-recognition could not be so important in females. Alternatively, it could be argued that perhaps chemical compounds in females’ scent might not show a level of interindividual variability that could allow this discrimination. However, in the second experiment, males were able to discriminate between different individual females, which strongly suggests that enough interindividual variation in the compounds found in the scent of females must occur.

We conclude that *T*. *wiegmanni* amphisbaenians have the ability to detect conspecific chemical stimuli and discriminate sex, partner of the pair and their own scent from those of other conspecifics, which suggests that chemical scents marks provide an important means of communication in amphisbaenians. Scent marks may contain sex-specific components that are employed in social chemical relationships. Further chemical characterization of these sex-specific odor will be necessary to identify active components that modulate social and reproductive behaviors. Finally, although chemosensory conspecific and sex recognition seem widespread among lizards, familiar, individual, and self- chemical recognition abilities have been less studied [23, 24]. Further studies could allow a phylogenetic comparison to test whether the amphisbaenians chemosensory abilities are simply similar to those of their closest phylogenetically related lacertid lizards or are rather more similar to those of ecologically closer lizard species with fossorial habits, such as some scincids, anguids, etc., as a result of adaptation to an underground life where vision is limited.
